# Effects of Extracorporeal Magnetic Stimulation in Fecal Incontinence

**DOI:** 10.1515/med-2020-0009

**Published:** 2020-01-30

**Authors:** Luigi Brusciano, Claudio Gambardella, Giorgia Gualtieri, Gianmattia Terracciano, Salvatore Tolone, Michele Schiano di Visconte, Ugo Grossi, Gianmattia del Genio, Ludovico Docimo

**Affiliations:** 1 Division of General, Mininvasive and Obesity Surgery, University of Study of Campania “Luigi Vanvitelli” Naples, via Luigi Pansini n° 5, 80131, Naples Italy; 2 Division of General, Mini-invasive and Obesity Surgery- Master of Coloproctology and Master of Pelvi-Perineal Rehabilitation. University of Study of Campania “Luigi Vanvitelli” Naples, Italy; 3 Department of Cardiothoracic Sciences - University of Campania “Luigi Vanvitelli”, School of Medicine, Naples, Italy; 4 Colorectal and Pelvic Floor Diseases Center, Department of General Surgery“S. Maria dei Battuti” Hospital Conegliano Italy; 5 Pelvic Floor Unit, I° Department of Surgery, Regional Hospital, Treviso, Italy

**Keywords:** Functional extracorporeal magnetic stimulation, Fecal incontinence, Pelvic floor rehabilitation, Magnetic chair

## Abstract

**Background:**

Fecal incontinence (FI) is a common condition that has devastating consequences for patients’ QOL. In some patients, the conventional functional pelvic floor electrical stimulation has been effective but is an invasive and embarrassing treatment. The object of the study was to evaluate the feasibility of functional extracorporeal magnetic stimulation (FMS) in strengthening the pelvic floor muscles without an anal plug and the embarrassment of undressing.

**Materials and Methods:**

Thirty patients (26 female and 4 males) with FI were enrolled. All patients were assessed during a specialized coloproctology evaluation followed by endoanal ultrasonography and anorectal manometry. All patients underwent an FMS treatment once weekly for 8 weeks. Patients’ outcome was assessed by the Cleveland Clinic Fecal Incontinence Score (CCFIS) and by the fecal incontinence QOL questionnaire (FIQL).

**Results:**

After 8 weeks, the number of solid and liquid stool leakage per week was significantly reduced (p<0.05) with a significant improvement of the CCFIS and of the FIQL (p<0.05). Moreover, the authors recorded a missed recruitment of the agonist and antagonists’ defecation muscles.

**Conclusion:**

FMS is a safe, non-invasive and painless treatment for FI. It could be recommended for selected patients with non-surgical FI to ensure a rapid clinical improvement.

## 1 Introduction

Fecal incontinence (FI) is defined as the involuntary loss of solid or liquid feces. It is a prevalent condition with unpleasant consequences for quality of life (QOL) [1]. FI can affect individuals of all ages; its overall prevalence in adults is calculated to be 11% to 15%, increasing with age QOL [2]. However, its real epidemiology appears to be underestimated as patients tend to avoid seeking medical care due to considerable embarrassment, social isolation, and stigma [3]. Different types of incontinence manifestations can range from unintentional elimination of flatus, soiling, to the complete loss of bowel contents, but in all cases it greatly impairs QOL. Mechanisms of continence involve a complex interplay between stool consistency, rectal compliance as reservoir, muscle groups of the pelvic floor, and proper function of the anal sphincter complex. Alterations in any of these elements, starting from stool consistency to muscular proficiency, may impact continence to flatus, liquid and solid stool, and arouse FI-connected symptoms. FI severity depends on the type and frequency of loss episodes, and of course, their role in modifying patients QOL.

In selected patients, functional pelvic floor treatment as electrical stimulation has been reported to be effective for FI as confirmed by several randomized controlled trials [4, 5, 6]. Its usefulness may be related to its unique role in inducing consciousness of the anal area, a feature that may be as useful as the stimulation itself [7, 8]. In this perspective, increasing sensitivity in those body areas might help to improve the specific recruitment of anal sphincters, thereby avoiding the inappropriate recruitment of agonists and antagonist groups of muscles. However, although it is an effective technique, electric stimulation can be considered an invasive and embarrassing treatment because of patients’ having to be undressed while undergoing rehabilitation and the necessity of insertion of an anal plug.

The aim of this study was to investigate the effectiveness of functional extracorporeal magnetic stimulation (FMS) as a non-undressed and no-probe-needed alternative technique to other functional therapies in patients affected by idiopathic FI, as an option for functional pelvic floor treatment.

## 2 Materials and methods

### 2.1 Patients and methods

Consecutive patients affected by FI and referred to a Teaching Hospital (Division of General, Mini-Invasive and Obesity Surgery - Master of Coloproctology and Pelvic Floor Rehabilitation, University of Campania “Luigi Vanvitelli”, Naples, Italy) between October 2018 to March 2019 were enrolled in the study. The local ethical committee approved the study protocol. The study was conducted in accordance with the Helsinki Declaration.

All patients were informed about aims, procedures and follow-up; participants also signed a written informed consent.

Selection criteria for the study were patient’s clinical history of FI for stool, liquid or flatus, and a Cleveland Clinic Fecal Incontinence Score (CCFIS) > 10. [9,10] Patients affected by urge incontinence were excluded from the study. The selection of patients with defecatory disorders amenable for rehabilitation treatment was obtained using a diagnostic protocol comprising proctologic examination, clinico-physiatric assessment, muscular synergies and instrumental evaluation [11].

All patients were assessed during a specialized coloproctology evaluation in a teaching hospital. A clinical examination was performed on all patients and information on bowel function, pregnancies, episiotomy, possible presence of pudendal nerve neuropathy, diabetes, and other associated diseases were recorded. Each patient underwent a physical examination to evaluate the resting anal tone and the muscular synergy: by inviting the patient to contract the anal sphincter, it was verified whether adductor (agonist muscles) or abdominal muscles (antagonist muscles) were contracted [11,12]. The CCFIS was then obtained in all patients. This scoring system, through 5 items (solid stool leakage, liquid stool leakage, gas leakage, pads use, lifestyle restriction) each graded from 0 to 4, allows objective evaluation of incontinence severity in patients with FI [9,10]. The Fecal Incontinence QOL Scale (FiQL) was submitted to all patients to evaluate the impact of FI on four aspects of patients’ QOL before the rehabilitation program and to rate the patients’ QOL improvement after treatment. The score consists of 29 questions ranging over different domains of the patient’s life: their lifestyle, their forced behaviour due to incontinence loss episodes, their personal perception of the disease, and finally, rating the subjective embarrassment caused by FI. In detail, a lower mean value of the domains corresponds to a worse clinical condition [13]. A 3D endorectal ultrasonography (3D EAUS) was performed before magnetic chair treatment, and a high-resolution anorectal manometry (HRAM) was performed both pre-and post-treatment.

### 2.2 3D Endorectal Ultrasonography (3D EAUS)

The device used to practice the 3D EAUS was BK Medical Flex Focus 800 fitted with a rotating 360° probe (model n.2050), covered by a cap filled with water. All the patients were placed in left lateral (Sims) position; the examination identified any lesion of the internal anal sphincter (IAS) and external anal sphincter (EAS) [14,15].

### 2.3 High Resolution Anorectal Manometry (HRAM)

HRAM was performed using a solid-state probe (ManoScan™ AR catheter, Medtronic, Minneapolis, USA). Pressure sensors were placed 1-cm apart from each other; each one counted 4 radially recording sensors for a total of 28 pressure sensors. Intrarectal and intra-balloon pressures were recorded by two additional pressure sensors placed at the probe’s distal end (TactArray; Pressure Profile Systems, Los Angeles, California). A disposable inflatable balloon was located at the catheter tip. The patient was positioned in left lateral (Sims) position during the exam; the probe was then positioned in the anal canal with at least one recording site in the distal rectum, and then the procedure was delayed a few seconds to guarantee the patient self-awareness of the probe’s presence. The anal canal length, anal resting pressures, maximum voluntary contraction, and rectal sensitivity were evaluated using Bioview Analysis software (Sandhill Sci) [16]. Evaluating rectal sensitivity consisted of assessing first conscious rectal sensation, maximum tolerate volume, and evacuation stimulus, having an increasing pressured balloon placed in the rectum. The patients was asked to communicate the volume of the balloon that caused its perception (first conscious rectal sensation), the one that caused the beginning of the evacuation stimulus, and the one that led to the urge need of defecation.

### 2.4 Design of the study

Every pelvic floor rehabilitation treatment session began with augmenting the patient’s awareness of the pelvic floor by showing them pictures of the different structures involved in continence to assist the patient to construct a proper muscles recruitment.

**Figure 1:**
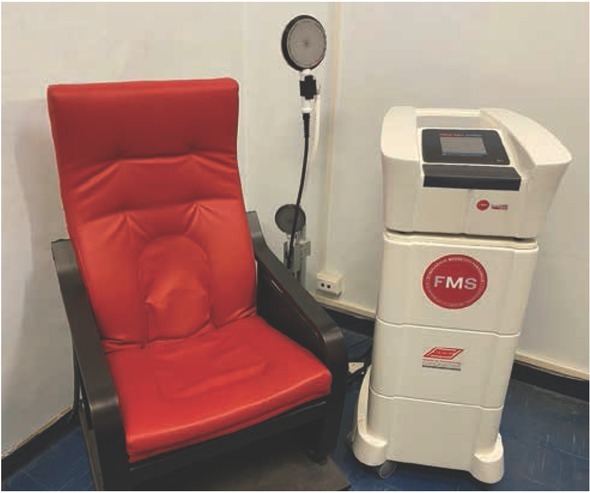
Tesla Care®Armchair for functional extracorporeal magnetic stimulation (FMS)

The device used was an armchair type magnetic stimulator FMS Tesla Care® (Max Medical Sassari – Italy) wherein the magnetic coil was placed in the bottom of the chair. [Figure 1] The stimulation was provided by an electromagnetic generator in the seat, controlled by an external unit. The intensity of the stimulation was 50–60 Hz, patients seated for an average time of 15 minutes on the chair, once weekly for 8 weeks. The magnetic field could be properly modified by the clinician by changing the frequency and amplitude on the generator. The perineum needed to be in the center of the seat because the highest power of the magnetic field corresponded to the center of the chair.

The treatment intervals were intermittent (5 seconds on, then 5 seconds off) to avoid muscle fatigue. The pulsed magnetic field generated by the device caused the contraction of the muscles of the pelvic floor without the need for an electrode. In detail, the rapid changes of the intensity of the magnetic field are generated by a phenomenon called electromagnetic induction, which is an electric current in neuronal cells. The depolarization of the latter passes distally to motor end plates and to the muscle fibers. The depolarization will also occur in sensory afferent fibers and autonomic nerves that may regulate local blood flow and other factors.

FMS treatment, while generating intermittent muscular contractions, increases the strength and endurance of the pelvic floor muscles. Additionally, the patient learns how to properly perform exercises that strengthen the muscles.

### 2.5 Follow up

After the 8 weeks treatment, the patients were clinically scored with CCFIS and instrumentally evaluated with HRAM. Moreover, the FIQL questionnaire was re-submitted to the patients.

### 2.6 Statistical analysis

Data were analyzed using the statistical package for social sciences (SPSS, version 16.0, Chicago, IL). Qualitative data are expressed as percent, and quantitative data are expressed as the means. Statistical significance was defined as p<0.05 with a confidence interval (CI) at 95%.

## 3 Results

Thirty patients met the aforementioned inclusion criteria, 26 females (86%) and 4 males (16%). Nine patients suffered of idiopathic FI (4 males and 5 females), 21 females had previously given birth, and 7 of them had undergone episiotomy reporting a I-II degree according to Sultan classification of perineal obstetric injury [17]. Overall mean age was 65 (range 38–74). Seven patients were affected by type 2 diabetes. Patient demographics are summarized in Table 1. Clinical and instrumental evaluations were appointed at the beginning of the treatment session. The mean CCFIS value before treatment was 12.4. The mean subscore values of the four domains of FiQL were, respectively, lifestyle 2.6, coping 2.0, depression 3.1, and embarrassment 1.8. Physical examination showed incorrect synergies in 21/30 of the patients (70%), involving buttocks, adductors, and abdominal muscles antagonist synergies only in 2/21 patients (10%). All features improved by the treatment are summarized in Table 2. Anal manometry showed a mean basal pression value of 46 mmHg (min 35 mmHg–max 65). Moreover, mean maximum voluntary contraction value was 110 mmHg, with an average duration of 14 seconds. Rectal sensibility was valued as 30 (25–35) mL, 60 (55–70) mL, and 120 (110–130) mL. Endoanal ultrasound demonstrated in 7/30 (23%) patients a lesion of the internal anal sphincter (transversal extension 35° mean (30–40), longitudinal extension 1 cm (0.8–1.4). Only 3/30 (10%) patients had lesions of the internal sphincter (transversal extension 40° mean (35–45), longitudinal extension 1.2 cm (1–1.6). None of the patients had concurrent lesions of internal and external sphincters.

**Table 1:** Patients demographics features. Values are expressed as number of cases or mean (*). FI (Fecal Incontinence)

	Patients group (n=30)
Age	65* (51-69)
Gender	26 females (86,7%)
	4 males (13,3%)
Idiopathic FI	5 females (16,7%)
	4 males (13,3%)
Past deliveries	21 (70%)
Previous episiotomy	7/21 (23,3%)
Comorbidities	7 (type II diabetes) (23,3%)

**Table 2:** Pre and post treatment clinical and instrumental parameters. Values are expressed as mean or number of cases (*). FMS (functional extracorporeal magnetic stimulation); CCSFIS (Cleveland Clinic Faecal Incontinence Score); FiQL (Fecal Incontinence Quality of Life Scale); HRAM (High Resolution Anorectal Manometry)

Item	Before FMS	After FMS	p
CCSFIS	12.4	4.7	<0.05
FiQL		3.2	
-lifestyle	2.6	2.7	<0.05
-coping	2.0	3.6	<0.05
-depression	3.1	2.4	<0.05
embarassment	1.8		<0.05
Buttocks/adductor synergies	21/30*	3/30*	<0.05

Abdominal synergies	2/30*	0/30*	NS

HRAM			
- basal pression	46 mmHg	48 mmHg	NS
- maximum voluntary contraction	110 mmHg	114 mmHG	NS
- contraction duration	14seconds	15 seconds	NS

**Figure 2:**
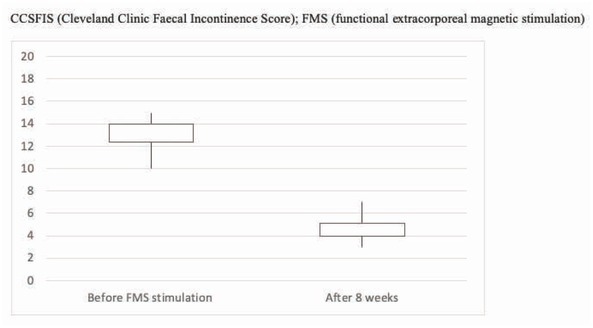
Modifications of the CCSFIS before and after FMS treatment

After treatment, a significant improvement of the CCIFS was recorded in 24 patients (80%). The mean CCIFS post-treatment value was 4.7, with a mean value reduction of 60% (p<0.05), its deducible clinical correlation was given by a statistically significant reduction of liquid and solid stool leakage per week. CCIFS modifications are represented in Figure 2 and Table 2.

Physical examination after treatment showed incorrect synergies in 3/30 of the patients (10%), involving buttocks and adductors, whereas abdominal muscles were stimulated in none of the patients. [Table 2]

**Figure 3:**
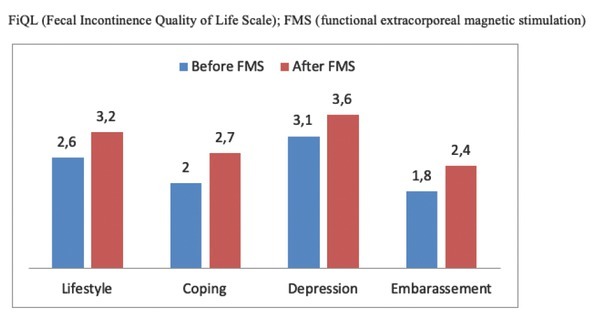
Modifications of the four domains of FiQL after FMS

FiQL domains scores improved in 27 out of 30 patients (90%). Post-treatment FiQL scores were 3.2, 2.7, 3.6, and 2.4 regarding lifestyle, coping, depression, and embarrassment patterns respectively. FiQL modifications are shown in Figure 3 and Table 3 showed the number of patients improved after FMS according to the clinical items.

**Table 3:** FI clinical items improvements. Values expressed as number of cases and percentage. FI (fecal Incontinence) FMS (functional extracorporeal magnetic stimulation); CCSFIS (Cleveland Clinic Faecal Incontinence Score); FiQL (Fecal Incontinence Quality of Life Scale); HRAM (High Resolution Anorectal Manometry)

Item	Patients improved after FMS	Percentage
CCSFIS	24/30	80%
FiQL	27/30	90%
Buttocks/adductor synergies	21/30	70%
Abdominal synergies	30/30	100%

**Figure 4:**
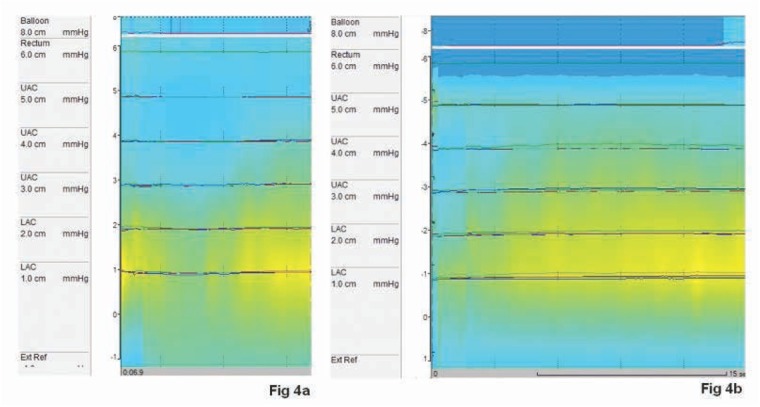
Pre FMS treatment (a) and post FSM treatment (b) HRAM (High Resolution Anal Manometry) showing no significant sphincteral improvement

No statistically changes were recorded in anal manometry after the eight weeks’ treatment time. In fact, the mean basal pression value was 48 mmHg (36–68 mmHg) and the mean maximum voluntary contraction value was 114 mmHg with an average duration of 15 seconds (p=NS) [Table 2] [Fig 4].

## 4 Discussion

Fecal incontinence is a multifactorial disease. Different mechanisms of continence are involved in the retention of gas and stools. Loss of continence can result from solitary anatomic defects, or more often, from alterations such as lesions or sphincters either caused by deliveries [18,19].

Hence, fecal incontinence is not only a diagnosis but a frequent and debilitating common final pathway symptom resulting from numerous different causes, regarding muscles competence, proper innervation, and the complex functioning of all these structures as a unit. That is, pelvic floor rehabilitation, having a primary target to recreate harmony among all the pelvic floor structures rather than curing symptoms, clearly has its important role in improving FI. The first step is to induce awareness of the pelvic floor muscles areas; the role of these areas in determining rehabilitation usefulness has exhaustively been studied [7].

Among other rehabilitation treatment techniques, anal electrostimulation is presently routinely performed and known as an effective nonoperative treatment for FI. It’s also been speculated that aside from the mere electric stimulation, this technique might owe its curative role to its ability to arouse the patient’s consciousness of the anal canal based on the fact that the sole knowledge of certain parts of the body better allows their selective recruitment [7]. Besides its usefulness, the extremely low compliance of patients to electrostimulation has also been assessed [20]. As a matter of fact, patients need to be undressed and experience the insertion of an anal plug; both details that strongly determine patients embarrassment and dis-comfort leading to the avoidance of this treatment. Therefore, the FMS is a relatively new technique that combines electrostimulation’s usefulness to a higher compliance. FMS represents a novel application of a classic principle of physics: a flow of electrons within the field will be induced by a changing magnetic field. The pulsing of the magnetic field precisely induces small currents to flow in the tissue that has the role of inducing depolarization of nerve axons, leading in turn to a nerve impulse travelling both in proximal and distal directions. The subsequent relapse of acetylcholine will result in the depolarization and contraction of the corresponding muscle fibers; the key to the efficacy of FMS is the depolarization of nerve fibers as it induces the strengthening of the muscles. FMS clinical effect is changing the activity of muscle groups in the pelvic floor. The repeated activation of a muscle’s activity caused by its nerves depolarization builds muscle strength and endurance. FMS is painless, and no electricity passes through the body, only a magnetic flux. Moreover, in functional electrical stimulation, a greater electrical current is needed to modulate the nerves because of the high impedance of the tissues and bones than demanded by FMS [21].

Different studies have assessed FMS effectiveness in urinary incontinence [22]. Yamanishi et al examined the effects of magnetic stimulation as well; they studied how FMS augmented urethral closure in healthy volunteers. During pelvic floor stimulation, they demonstrated and recorded significant increases in maximum urethral closure pressures [23]. These above-mentioned principles studied in urinary incontinence are applicable also to fecal incontinence, although literature examining them is still meager. To the best of our knowledge, the current study is the first reporting the effectiveness and feasibility of FMS in the treatment of FI.

The present study included patients with idiopathic FI. In comparison with the baseline value, a statically significant improvement in CCFIS score was recorded: it was found to be decreased after the treatment period, with a mean value reduction of 60%, and an absolute mean value of 4.7. FMS clearly improved gas, liquid, solid stool leakage, and improved the satisfaction of all patients in our cohort, according to a statistically significant increase of all the four domains of FiQL values (p>0.05). This result attests the decrease of psychological distress related to the patients’ clinical condition as well as a reduction of the forced everyday life changes imposed by the pathology. In our series we did not observe a significant improve in of basal pressure, maximum voluntary contraction, and duration of contraction at HRAM. This is apparently in contrast with the clinical parameters improvements recorded in our cohort. Most likely, the clinical symptoms relief is associated to a better recruitment of antagonist and agonist muscles, to the increase of strength and endurance of the pelvic floor muscles, and to the awareness of the pelvic floor itself acquired by the patients.

Therefore, compared to anal electrostimulation, FMS increases major patient compliance as it is painless, not invasive, and does not requiring undressing. FMS creates neural stimulation which can penetrate into all kinds of tissue with no attenuation; therefore, intensity of the energy is lower than anal electrostimulation, drastically reducing pain. Given lack of invasiveness, FMS might be particularly suitable for children as well, even if there a lack of results in literature remains. On the other hand, other kinds of conventional electro stimulations have been studied in children affected by pediatric voiding dysfunction, which have proved effective but uncomfortable. Undressing for attaching electrodes at every stimulation session can, in fact, make children nervous and noncompliant, and the required electrical stimuli for actual neural stimulation causes more pain and discomfort in children than in adults [24]. Yokoyama et al reported that frequencies of 20 to 50 Hz were effective for stress urinary incontinence and significantly increased the maximal intraurethral pressure [6]. Presently, no study reports the optimal pulse duration and frequency of FMS in FI. In our series, we adopted a frequency of 50–60 Hz for 15 minutes on the chair, once a week, for 8 weeks. A drawback of the FMS treatment is certainly the cost. FMS, in fact, is an expensive method with the chair cost that is about 40.000 € [21]. Contrary to most authors [6,21,24], Voorham et al in their study of 74 patients affected by urinary incontinence, reported no differences in pelvic floor muscle activity, pad-test, QoL, voiding diary, and urodynamics in patients treated with FMS. Those authors underlined the importance of patient selection and concluded that ‘the chair’ is suitable to train awareness of the location of the pelvic floor, but the need for active pelvic floor muscle exercises remains [4].

The present study has some limitations. First is the small sample size, which precluded any analysis of the effect of covariates. In addition, the real question of neuromodulation is the long-term recurrence of symptoms when the neural stimulation treatment stops [25,26]. We do not yet have long‐term follow‐up data after stimulation, as the present study was prospective and focused on assessing FMS effectiveness and feasibility. Moreover, different programs of stimulation can be used by the physician to increase effectiveness and likely to reduce recurrence of the symptoms; thus studies on the appropriate duration of stimulation, combined treatments, and randomized controlled studies with a sham‐stimulation group are needed.

## 5 Conclusions

FMS of the pelvic floor is an effective treatment for idiopathic fecal incontinence, resulting in patients improved QOL and decrease of incontinence scores, comparable to conventional anal electrostimulation. Extracorporeal magnetic stimulation is a comfortable technique as it avoids patient embarrassment through no need to get undressed; it also eliminates the discomfort and invasiveness related to the anal probe. Further long-term and comparative studies are needed to investigate the efficacy of the treatment in a large population with pelvic floor dis-orders.
